# Total Intravenous Anesthesia for Intraoperative Neurophysiological Monitoring in a Child With Diastematomyelia: A Case Report

**DOI:** 10.7759/cureus.75233

**Published:** 2024-12-06

**Authors:** Theyjes Sabesan, Ramamurthy Balaji, Manoj Vishak, Rajesh Priyadharshini, Jalaludeen Samsudeen

**Affiliations:** 1 Anaesthesia, SRM Medical College Hospital and Research Center, Chennai, IND; 2 Radiology, SRM Medical College Hospital and Research Center, Chennai, IND

**Keywords:** intraoperative neuromonitoring (ionm), motor evoked potentials (meps), somatosensory evoked potentials (sseps), spine surgery anesthesia, split cord malformation, total intravenous anesthesia (tiva), type 2 diastematomyelia

## Abstract

Intraoperative neurophysiological monitoring (IONM) has achieved popularity because it facilitates monitoring of the functional integrity of neural structures under general anesthesia. It aids in the early detection of injury and minimizes postoperative neurologic deficit or neurologic morbidity from surgical manipulations of various neurologic structures. The patient mentioned in this case report presented with lower limb radiculopathy and was diagnosed with diastematomyelia Type II, and she was planned for surgical intervention under general anesthesia. Preoperatively, a team of surgeons, anesthetists, and neurophysiologists must discuss modalities of IONM to be used, expected changes, and alarm criteria. Anesthesia drugs need to be appropriately selected to facilitate IONM, as they affect the somatosensory evoked potential (SSEP) and motor evoked potential (MEP) responses obtained. We have facilitated IONM by avoiding muscle relaxants and inhalational agents for this specific patient. Risk-benefit must be assessed before the selection of a patient for evoked potential (EP) monitoring, as it may be rarely associated with complications such as burns, seizures, bite injuries to the lips, tongue, and endotracheal tube. Teamwork with meticulous planning, preparation, and multidisciplinary communication is essential for the safe conduct of spine surgeries with SSEP and MEP monitoring. In this case report, we discuss various considerations for anesthesia management in a patient with diastematomyelia undergoing spine surgery with intraoperative EP monitoring.

## Introduction

Diastematomyelia, or split cord malformation, is a rare congenital condition where the spinal cord is split by a septum made of osseous, cartilaginous, or fibrous tissue. It can be classified into two types: Type I, with two dural sacs and an osseous or cartilaginous septum, and Type II, where both hemicords are in a single dural sac separated by a fibrous septum. Surgical or medical management may be necessary, especially in cases with progressive neurological symptoms. This case report discusses a patient with lumbar diastematomyelia Type II, presenting with lower limb radiculopathy and treated with surgery involving laminectomy, untethering of the cords, and resection of the septum. Although such surgeries carry a risk of neurological deficits, intraoperative neurophysiological monitoring (IONM) can help minimize risks by detecting injury early. IONM aids in assessing neural structures such as the cerebral cortex, brainstem, spinal cord, and peripheral nerves. It is particularly useful during spinal deformity surgery, where there is a high risk of spinal cord injury. The report also addresses the anesthetic considerations during surgeries that involve IONM.

## Case presentation

An 11-year-old female child, with complaints of pain radiating to the right lower limb, associated with numbness, presented electively for laminectomy, untethering of cords, and resection of the septum under general anesthesia. A diagnosis of Type II diastematomyelia was made by the neurosurgery specialists through an MRI of the lumbosacral spine.

**Figure 1:**
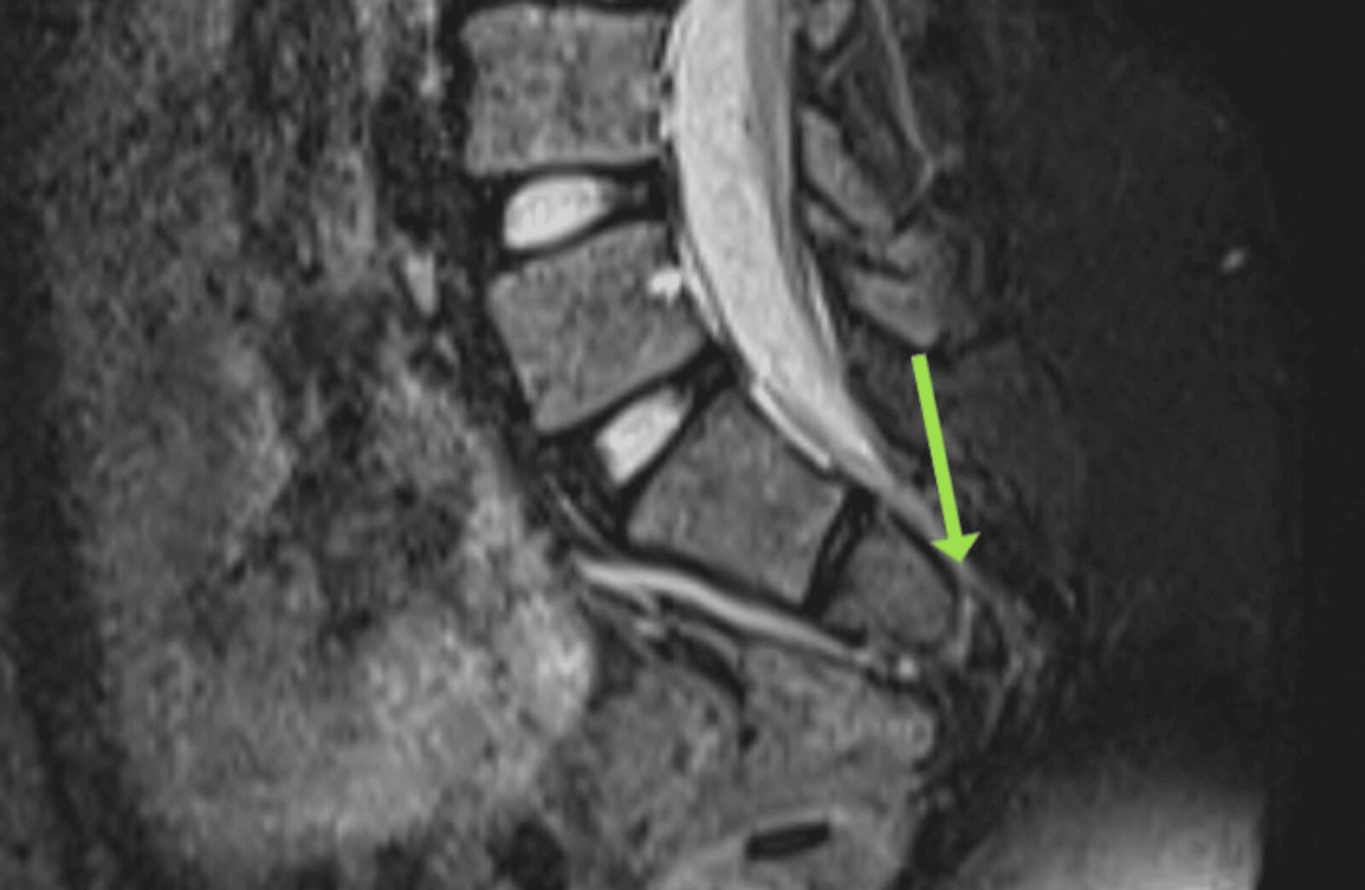
MRI image The arrow shows caudal agenesis.

**Figure 2:**
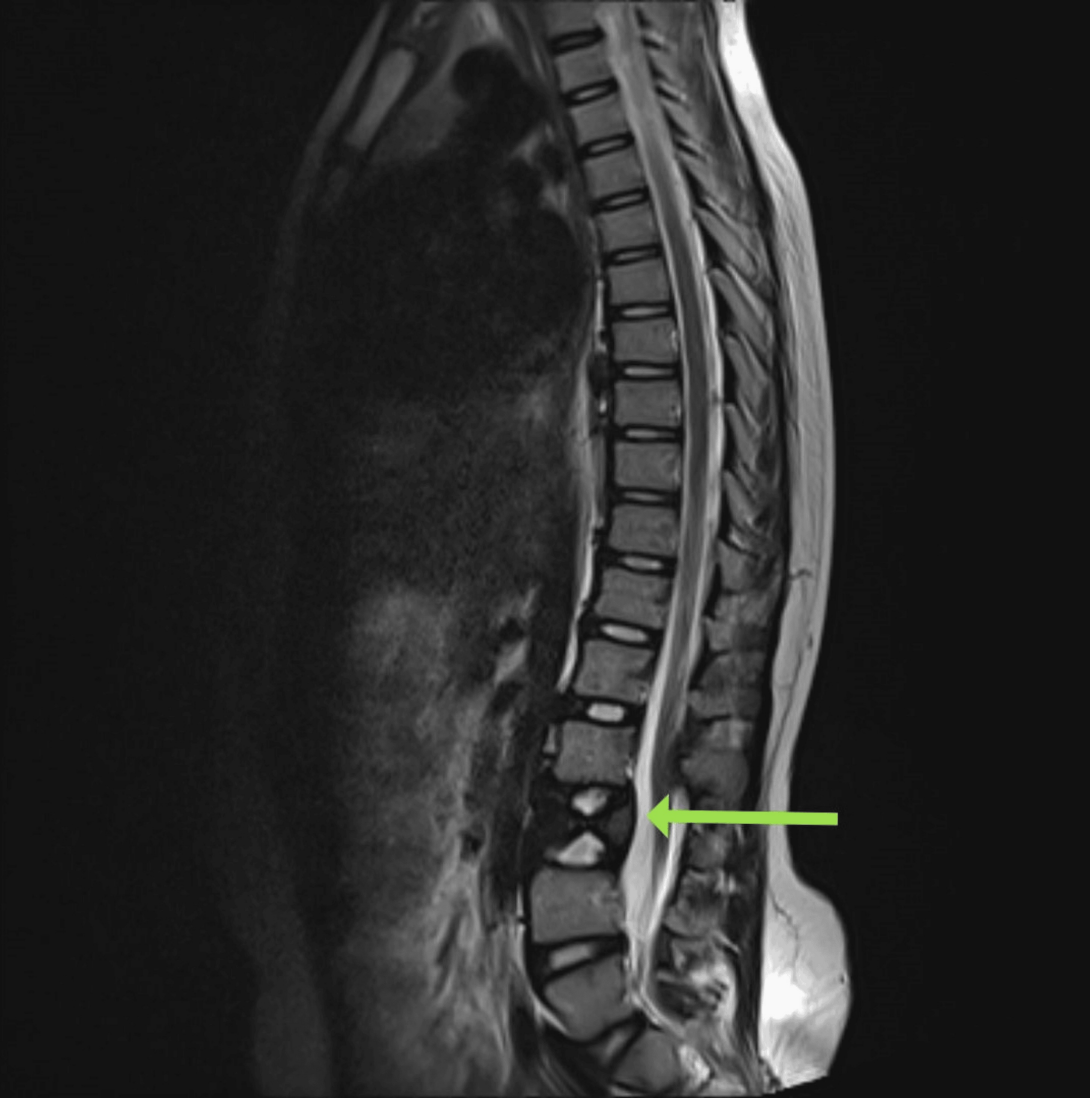
MRI image The arrow shows the L2 hemivertebra in the sagittal section. At the level of the L1 vertebra, the cord appears adherent to the posterior dura-tethered cord.

**Figure 3:**
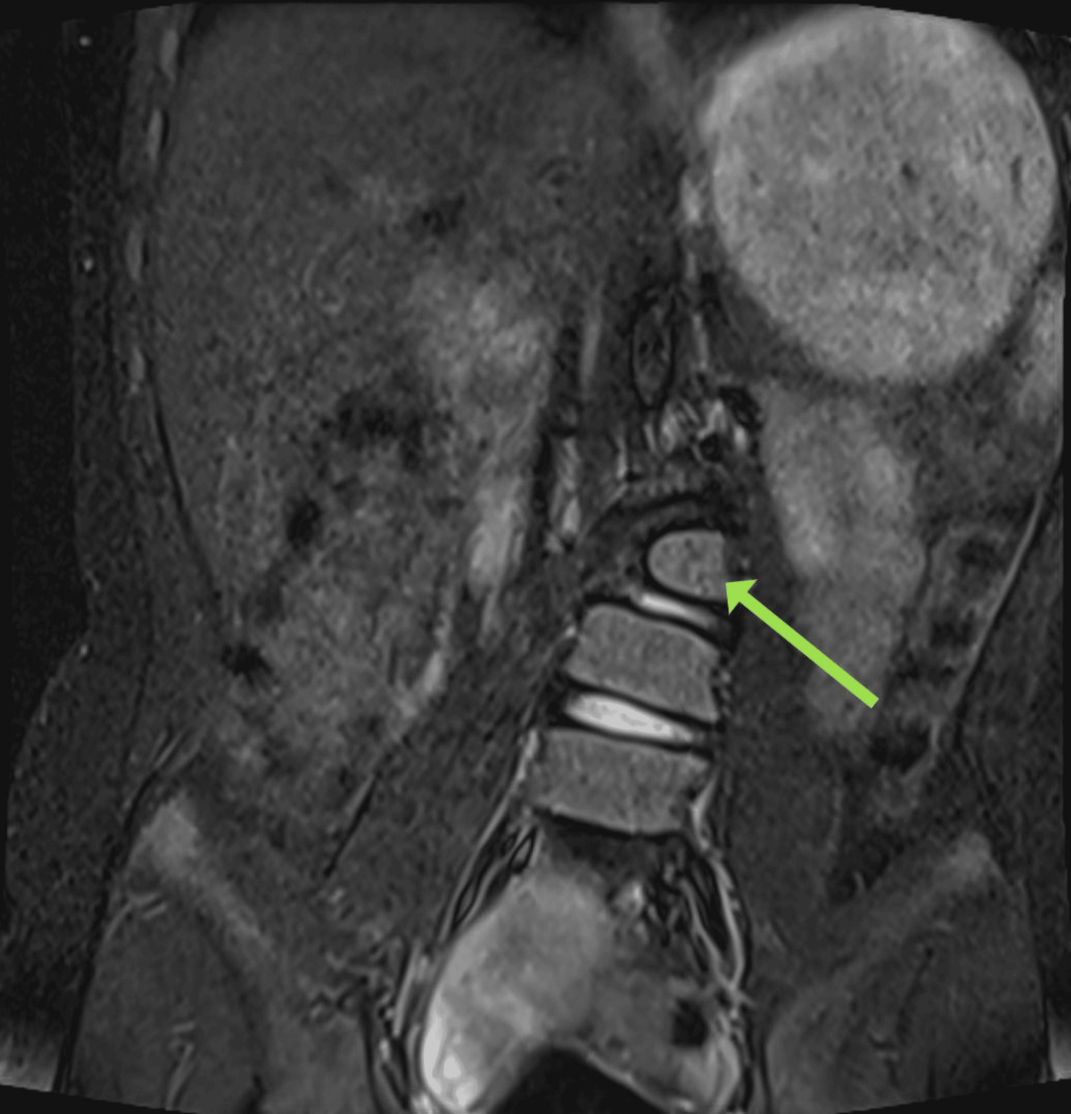
MRI image The arrow shows the L2 hemivertebra in the coronal section with levoscoliosis (Cobb's angle of about 25%).

The MRI findings revealed significant spinal anomalies. There was a butterfly vertebra and lumbosacral transitional vertebra present. Additionally, two hemicords were observed within a common dural sac at the L1-L2 and L3-L4 levels, but no evidence of osteocartilaginous spurs or hydromyelia was noted, indicating diastematomyelia Type II. There was also agenesis of the S4 and S5 vertebrae and the coccyx, consistent with caudal regression syndrome (Figure 1). The conus medullaris appeared low-lying, terminating at the L3-L4 level. The cord was adhered to the posterior dura at the L1 level, indicating a tethered cord (Figure 2). The Cobb's angle at the L2 hemivertebra measured approximately 25% (Figure 3). Furthermore, there was non-fusion of the spinous processes of the L2, L3, and L5 vertebrae, along with non-visualization of the posterior elements of the sacrum from S1 to S3.

The child had undergone congenital talipes equinovarus (CTEV) correction surgery at 3 months of age and has abnormal gait because of it. She also had an isolated episode of seizure at 9 months of age, her past medical history was otherwise uneventful.

Upon examination, the patient weighed 40 kg, was able to walk without assistance, and had no cognitive dysfunction. She had right lower limb radiculopathy and an abnormal gait due to scoliosis and CTEV. Her preoperative cardiac assessment, airway assessment, and vital signs were normal. Her blood investigations such as complete blood count, renal function test, coagulation profile were also within normal limits.

On the day of surgery, peripheral lines were secured in both her upper limbs with 20G venflons and standard ASA monitors were attached. Electrodes were placed in the parietal region of the scalp corresponding to the motor and sensory areas, and when there were changes in the waveforms, it indicated that the patient was emerging out of anesthesia and the rate of infusions had to be increased accordingly.

**Figure 4:**
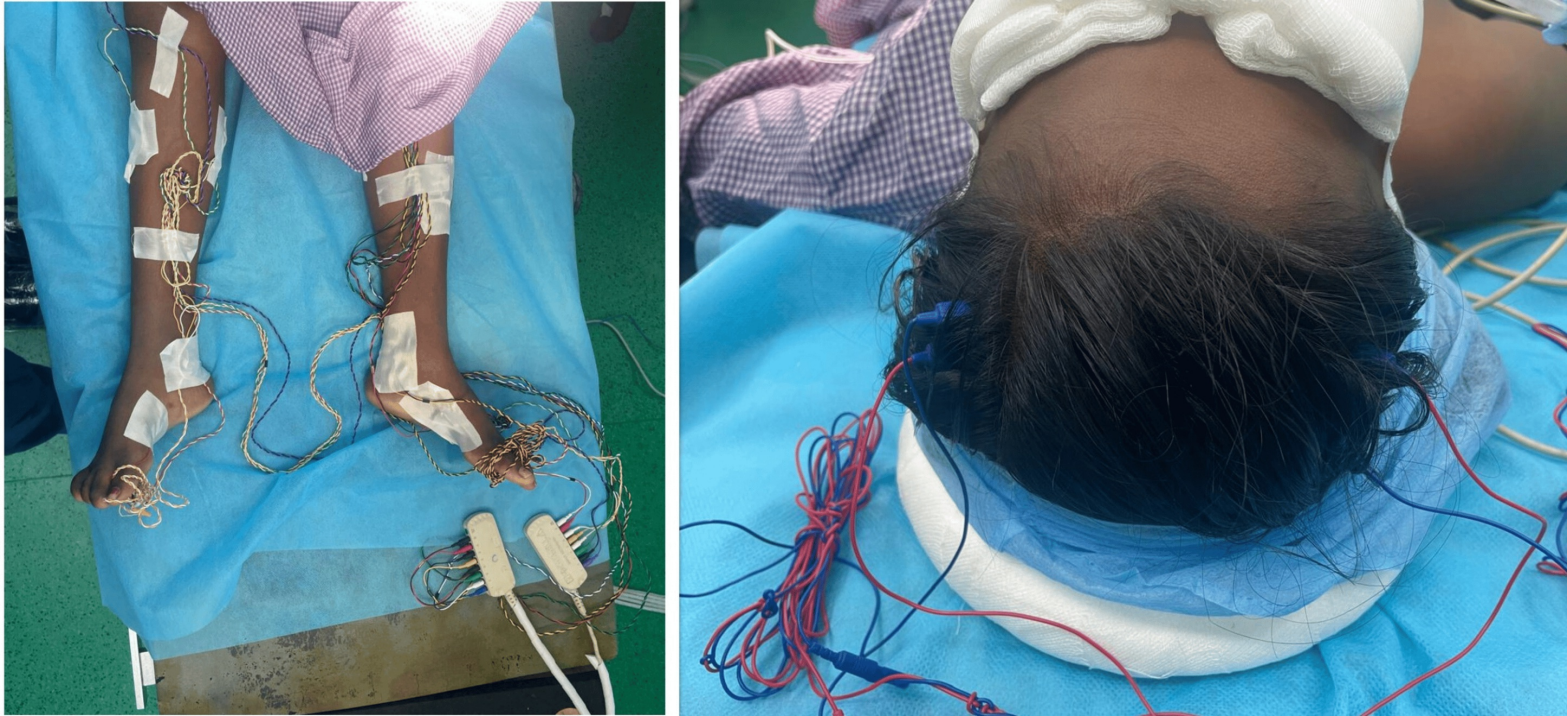
Electrode placement for IONM Left: Electrodes in the lower extremities Right: Electrodes in the head IONM, intraoperative neurophysiological monitoring

**Figure 5:**
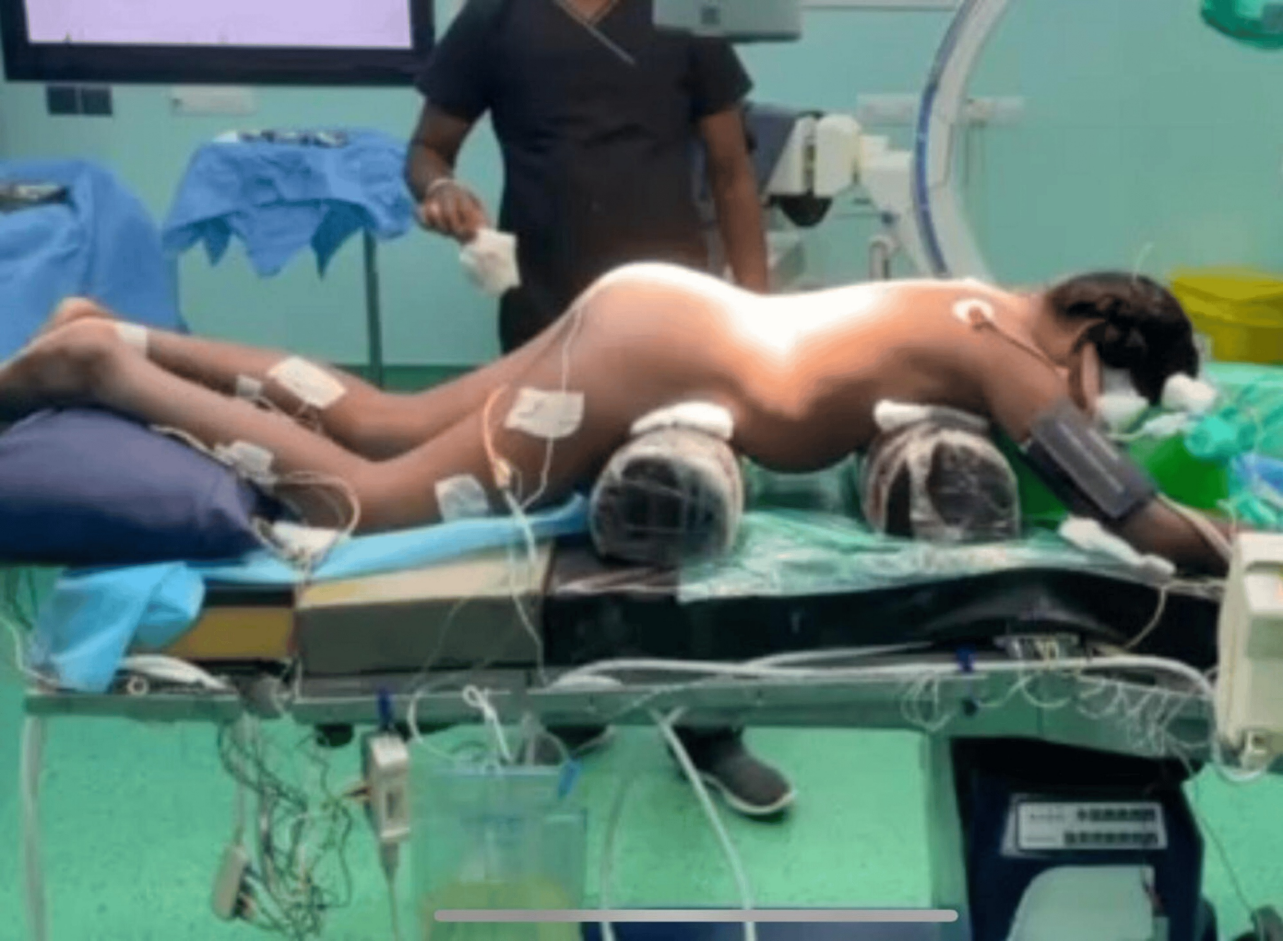
Position of the patient during the procedure Pressure points were padded adequately, and the abdomen was kept free.

**Figure 6:**
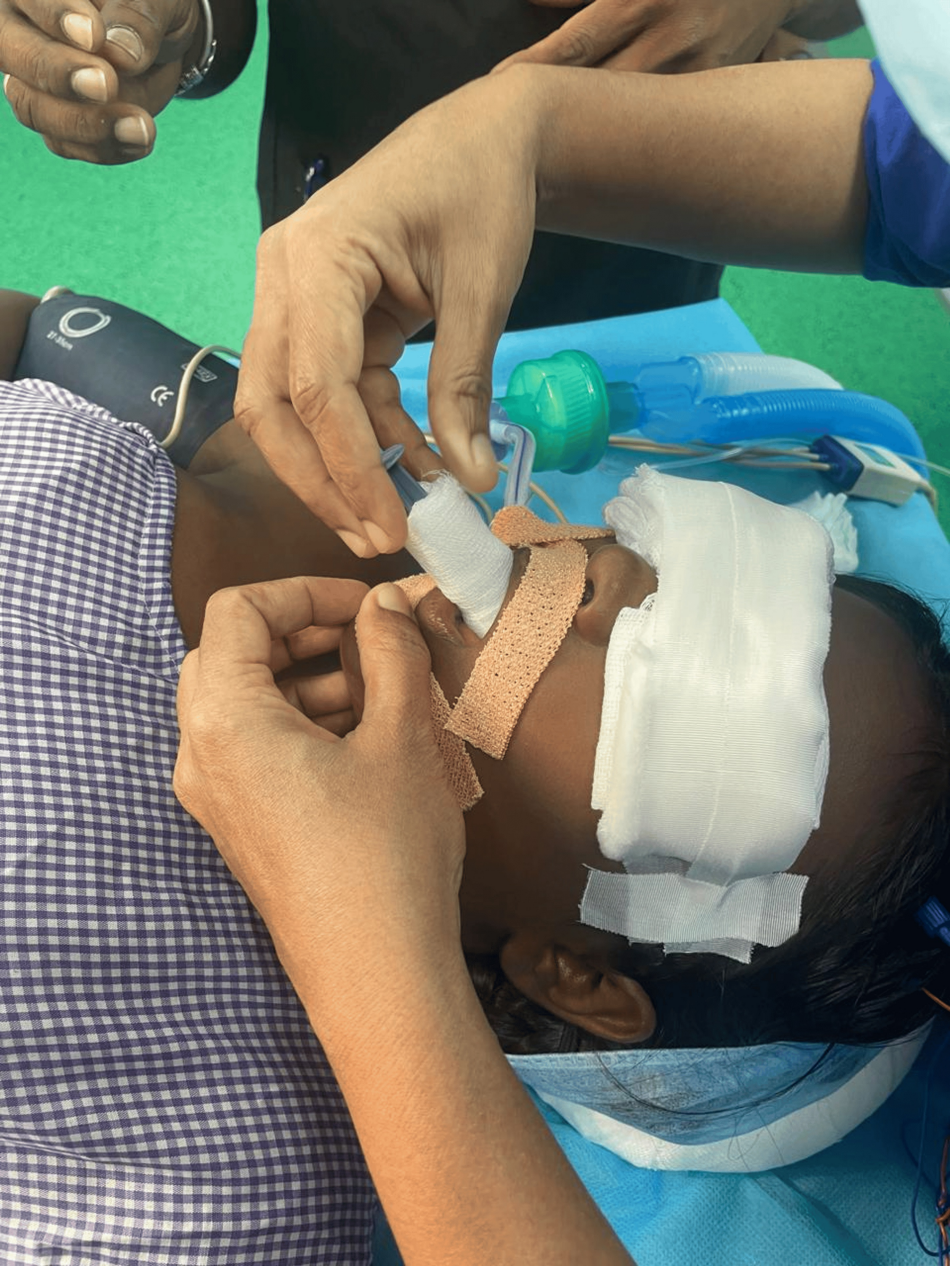
Bite block

Also electrodes were placed in the extremities (right and left hands, B/L quadratus muscles, B/L tibialis anterior muscles, B/L gastrocnemius muscles), and when there was a deviation from the normal baseline waveform, it indicated that there was surgical manipulation. Thus intraoperative neuromonitoring was facilitated (Figure 4). We also had other objectives such as preventing hypothermia, hyperthermia, hypoxia, and hypotension, as they could alter the evoked potentials (EPs). Patient was pre-medicated with glycopyrrolate (0.2 mg), midazolam (1 mg), and ondansetron (4 mg). After preoxygenation, anesthesia was induced with propofol (3 mg/kg) and fentanyl (3 mcg/kg). Muscle relaxants were avoided. Bag-mask ventilation was easily achieved without the requirement of oropharyngeal airway. After induction, under aseptic precautions, transtracheal injection of 4% lignocaine (2 mL) was given, which was followed by two puffs of 10% lidocaine on the vocal cords and epiglottis. A size 6 endotracheal tube (ETT) was inserted and fixed at 17 cm. Patient was ventilated using an oxygen-air mixture (at 1-2 L/min) in volume control mode, delivering a tidal volume of 350 mL to the patient, with a positive end-expiratory pressure of 5 cm H_2_O, and maintaining peak airway pressures below 19 cm H_2_O. Adequate padding was given for the eyes and the patient was turned prone with appropriate padding, while keeping the abdomen free (Figure 5). A soft bite block was inserted to prevent damage to the teeth (Figure 6).

Anesthesia was maintained with total intravenous agents. Infusions of dexmedetomidine (0.3 mcg/kg/h) at 6 mL/h and propofol (8 mg/kg/h) at 14 mL/h was started, supported by boluses of fentanyl at frequent intervals in low doses, thereby preventing any intrusion in IONM recording. Muscle relaxants and sevoflurane were avoided to facilitate neuromonitoring. Adequate plane of anesthesia was maintained with the agents, and appropriate somatosensory EPs (SSEPs) and motor EPs (MEPs) were recorded as a part of IONM. Surgery lasted for about 7 hours; the patient remained hemodynamically stable without any untoward complications.

Near completion of surgery, infusion agents were weaned and eventually stopped. Patient was turned supine, and after adequate spontaneous ventilation, she was extubated and shifted to post-anesthesia care unit for postoperative monitoring after ensuring stable vitals in the immediate postoperative period.

## Discussion

**Figure 7:**
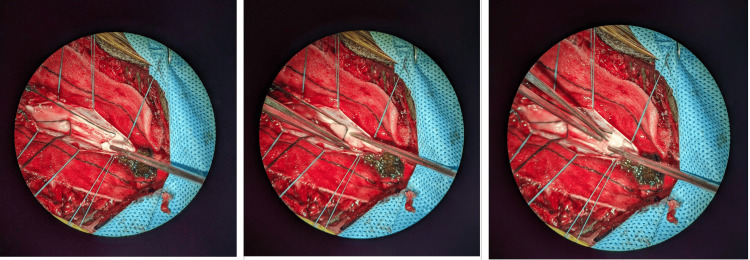
Surgical field through the microscope

During spine surgery, as Lall et al. mentioned, there is a risk of damage to the neural structures, which may result in postoperative neurological deficit (Figure 7) [1]. During general anesthesia, the functional integrity of the neural structures may be monitored using IONM. IONM may aid in the early detection of any neurologic damage to prevent postoperative neurological deficit [2]. IONM is crucial for surgeries that either risk disrupting the spinal cord’s blood supply or involve manipulation of the spinal cord, such as spinal tumor excision, scoliosis correction, spinal stenosis surgery, and interventions for spinal cord injuries [3,4]. In scoliosis surgeries, the incidence of neurologic complications can be as high as 3.7%-6.9%, which can be decreased to 0.5% with multimodal IONM [5]. 

IONM

EPs are one of the modalities used for IONM during spine surgery. IONM during spine surgery involves monitoring both the sensory and the motor tracts in the spinal cord.

EPs are a measurement of the electrical potentials produced in response to stimulating the nervous system (evoked) by sensory, electrical, or magnetic stimulation [6]. Responses to the sensory and motor stimulation cause transmission of neural signals, which can be recorded at various points along the pathway [7]. SSEPs and MEPs aid in monitoring the sensory and motor tracts in the spinal cord, respectively.

**Figure 8:**
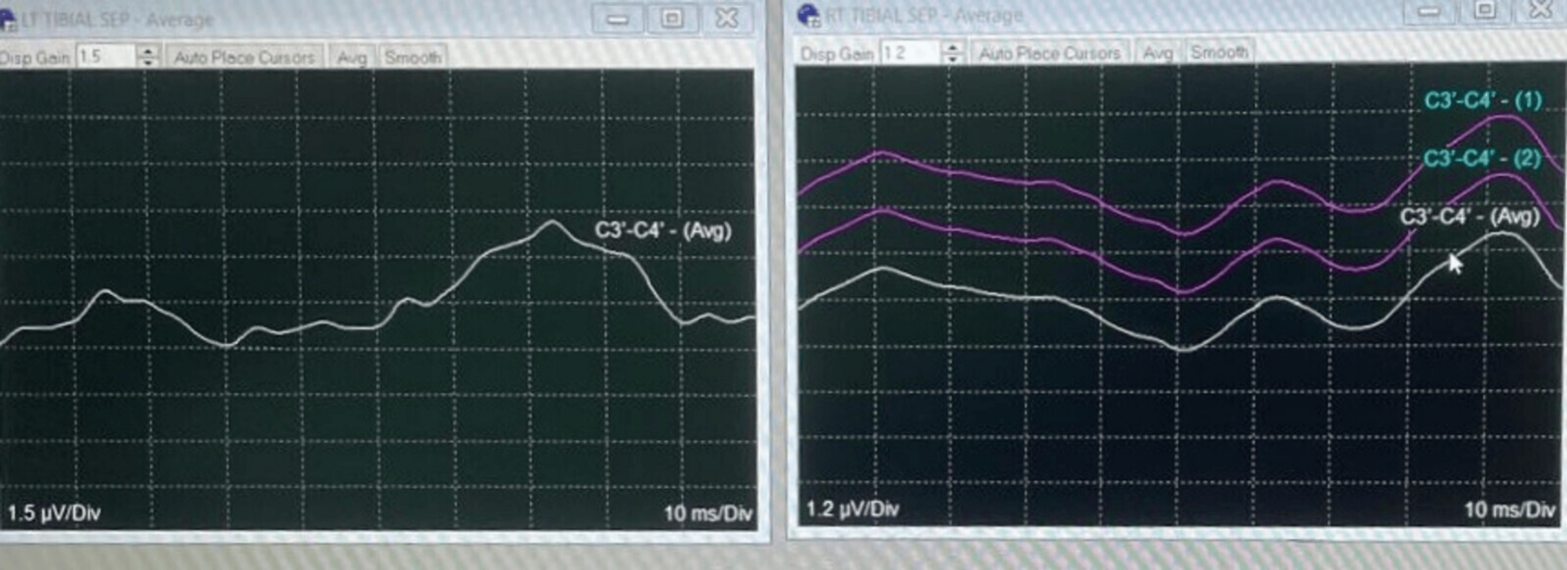
IONM Normal baseline waveforms IONM = Intraoperative neurophysiological monitoring

SSEPs are recorded by stimulation of the peripheral nerve on the extremities, with a recording of signals along the ascending sensory pathway. Commonly monitored nerves include the posterior tibial nerve, the peroneal nerve of the lower extremity, and the ulnar and the median nerve of the upper extremity [1]. The normal baseline waveforms of this case are shown in Figure 8. SSEPs are monitored continuously. Due to the low amplitude of SSEP responses, averaging is required, and identifying a meaningful change may take 3-5 minutes [8]. A decrease in amplitude greater than 50%, an increase in latency by 10% or more, or a combination of both suggests injury to the large fiber dorsal column pathways [9]. Various studies concluded that “SSEP monitoring is highly specific but weakly sensitive for postoperative neurological deficit following spine surgery” [10,11]. The main limitations include delayed signal detection, monitoring of only the dorsal sensory tracts of the spinal cord, and sensitivity to anesthetics and physiological factors [12].

**Figure 9:**
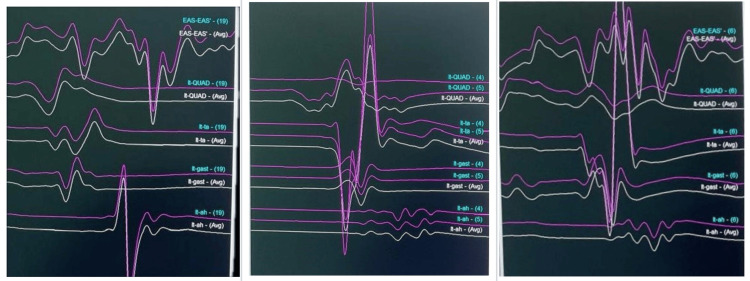
IONM Waveforms show alterations in the specific tract due to surgical manipulation. IONM = Intraoperative neurophysiological monitoring

The corticospinal tract is the primary direct route for signals from the cortex to the spinal cord and is critical for voluntary movement [3]. MEP recordings are made through transcranial electrical stimulation, with electrodes positioned on the scalp. The stimulation targets the pyramidal cells of the motor cortex, creating a depolarization wave that moves along the corticospinal tract and produces a direct (D) wave. Additionally, the activation of internuncial pathways in the cortex gives rise to a series of smaller indirect (I) waves [3]. Summation of D and I waves stimulates the peripheral nerve to produce compound muscle action potential. Muscle contraction (muscle MEP) is commonly used to monitor MEP [3]. Significant change in MEP may be assessed with the “threshold criteria,” the “amplitude criteria,” or “all or none criteria” [3]. The muscle MEP are of high amplitude; hence, averaging is not required to obtain response. Unlike SSEPs, MEPs provide information without delay, as averaging of responses is not required. EP may be affected by any drug or physical factor that may influence the electrical conduction along an axon [13]. Figure 9 shows the disturbance in waveforms due to surgical stimulus.

Preoperative evaluation

All patients should undergo a thorough preoperative evaluation, including screening for any contraindications to IONM such as loose teeth, epilepsy, proconvulsant medication, intracranial electrodes, vascular clips, shunts, cardiac pacemakers, or any other implanted medical devices. Any pre-existing neurological defects should be documented to facilitate intraoperative and postoperative surveillance and detection of new injuries.

Effect of various anesthetic agents on EPs

 Halogenated agents increase latency and decrease amplitude of cortical SSEPs in a dose-dependent manner, with isoflurane being the most potent and halothane the least. Due to varying patient responses, inhalational agents may be inadequate during IONM, leading to a preference for total intravenous anesthesia (TIVA). Propofol, which decreases cortical response amplitude but allows for quick recovery, is popular in TIVA, especially when combined with opioids, which minimally affect SSEPs. Opioids such as fentanyl have slight effects on SSEP amplitude and latency. Muscle relaxants must be carefully controlled to avoid excessive blockade, and any changes in SSEP or MEPs during surgery necessitate immediate team review and intervention. A checklist is recommended to ensure proper response to monitoring changes [14-20]. The effect of various anesthetic agents is summarized in Table 1. 

**Table 1:** Effect of intravenous drugs on evoked potentials *Without nitrous oxide, as mentioned by Ali et al. [3] A = Amplitude, L = Latency, MAC = Minimum alveolar concentration, MEP = Motor evoked potential, SSEP = Somatosensory evoked potential, − = Negligible or no effect, + = Minimal effect, ++ = Significant effect, +++ = Profound effect

Anesthetic agent	SSEP	MEP
Sevoflurane (1.5 MAC)*	↓A ↑ L	++
Nitrous oxide (60%-70%)	↓A - L	++
Propofol	↓A	++
Opioids	↓A ↑ L	-
Benzodiazepines	-	+++
Dexmedetomidine	-	+
Neuromuscular blockers	-	+++

Effect of physiological parameters on EPs

Physiologic parameters such as temperature, blood pressure, hematocrit, oxygenation, and carbon dioxide affect EP recording. Knowledge of these effects is essential to maintaining stable physiologic conditions throughout surgery. It is briefly summarized in Table 2, which helps to rule out any possibility of changes in recording attributed to these physiologic alterations.

**Table 2:** Effect of physiological factors on evoked potentials A = Amplitude, L = Latency, SSEP = Somatosensory evoked potential [3]

Physiological factors	SSEP
Hypothermia	↑L
Hyperthermia	↑A ↑L
Hypoxia	↓A ↑L
Hypotension	↓L
Hemodilution	↓A ↑ L
Hypocapnia	↓L

Positioning

The prone position is commonly used for spine surgeries. After intubation and attachment of all monitors, the patient should be placed in the prone position. However, it comes with its own set of complications such as raised intra-abdominal pressure, pressure sores, nerve palsies, postoperative visual loss, oropharyngeal swelling, and ETT dislodgement. In order to avoid these complications, we must secure the ETT properly and provide adequate padding to all pressure points. We must also place bolsters to ensure that the abdomen is free, with no undue pressure, and the head must be supported on a pillow with a cut-out, horseshoe, or in-head pin holder.

 Positioning itself may sometimes cause neurologic or vascular compromise affecting IONM. Patients who are at high risk of injury during positioning may need IONM performed in a supine position followed by monitoring in a prone position. This may detect any change in EP due to positioning, facilitating immediate correction to prevent damage due to positioning.

Safety and complications of EPs

Transcranial stimulation poses several risks due to the high voltage (600-900 V) and current used. One potential complication is bite injuries, which occur when the current stimulates the trigeminal nerve, leading to jaw contractions. This can result in patients biting their tongue or lips, with an incidence of 0.2%. Mandibular contractions may also cause patients to bite through an endotracheal tube, creating emergencies. Instead of using hard bite blocks, it is safer to use large cotton wads between the molars.

Seizures can be triggered by brain stimulation, so stimulation frequency should be limited in patients at risk; however, the risk of seizures with pulse train stimuli is low and has not resulted in morbidity.

Cardiovascular complications may arise if the current penetrates the hypothalamus or brainstem, and electrical artifacts can mimic cardiac arrhythmias. Therefore, transcranial stimulation should be avoided in patients with implanted defibrillators unless there is a significant risk of motor injury.

Postoperative care

At the end of surgery, it is desirable to have a rapid return of consciousness and extubation of the patient. Anesthetic drugs must be titrated in such a way that it will facilitate early awakening, which can help with immediate assessment of neuromuscular function and detection of any postoperative neurological deficit. The patient must be provided with multimodal analgesia in the form of local infiltration at the surgical site, intravenous paracetamol, nonsteroidal anti-inflammatory drugs, and/or opioids to ensure a pain-free, comfortable experience. Erector spinae block provides effective analgesia after spine surgeries.

## Conclusions

In reporting this case, we have explained the considerations involved in providing a good depth of anesthesia while facilitating IONM. IONM with SSEP and MEP is widely used during spine surgery to minimize the risk of postoperative neurological deficit. Both SSEP and MEP may be affected by anesthetic agents and physiological parameters. The anesthetist must formulate the anesthesia plan, considering the patient’s condition, procedure planned, modalities of IONM to be used, and expected changes in IONM, if any. TIVA, or balanced anesthesia technique, may be used to maintain anesthesia. It is essential to maintain stable anesthesia and physiologic parameters to rule out any change in EP due to these conditions. Appropriate care must be taken to avoid complications, and if they happen, then diagnose and treat them early to have better patient outcomes.
